# Presence of diabetic risk factors within a sample of community members in Windhoek, Namibia

**DOI:** 10.4102/phcfm.v16i1.4608

**Published:** 2024-11-12

**Authors:** Michelle C. Delpaul, Craig Vincent-Lambert

**Affiliations:** 1 Department of Complementary Medicine, Faculty of Health Sciences, University of Johannesburg, Johannesburg, South Africa; 2 Department of Emergency Medical Care, Faculty of Health Sciences, University of Johannesburg, Johannesburg, South Africa

**Keywords:** diabetes mellitus, metabolic, screening, risk factors, random blood glucose, non-communicable diseases

## Abstract

**Background:**

Non-communicable diseases (NCDs) continue to pose a significant challenge to global healthcare systems. In African contexts, diabetes ranks among the top four NCDs. The disease often goes undetected because patients are commonly asymptomatic in the early stages. Screening, early diagnosis and patient education may prolong life and reduce costs associated with managing the disease.

**Aim:**

To identify risk factors for diabetes among community members in Windhoek.

**Setting:**

Data were gathered during a health-screening outreach programme in Otjomuise, a peri-urban settlement near Windhoek, Namibia.

**Methods:**

An observational analytical cross-sectional study design was used, measuring weight, height, abdominal girth, random blood glucose (RBG) levels, lipid counts, and blood pressure in 342 participants. Descriptive analysis determined the percentage of participants with abnormal measurements indicating diabetes risk.

**Results:**

Analysis showed that 77% of participants had multiple risk factors for diabetes. Specifically, 67% had elevated RBG levels, 58% had elevated blood pressure, 45% had elevated body mass index, and 26% had raised lipid counts.

**Conclusion:**

These findings align with existing literature, which shows a significant portion of community members have undetected risk factors that may predispose them to the development of diabetes. Early detection, targeted interventions and ongoing monitoring for individuals with risk factors can save lives and reduce costs. Larger-scale research is necessary to better quantify the prediabetic population across various African contexts.

**Contribution:**

This study highlights the urgent need for early diabetes detection and intervention strategies in diverse African communities.

## Introduction

Non-communicable diseases (NCDs) are the leading global cause of death, accounting for 74% of deaths worldwide.^1^ Diabetes mellitus (DM) is one of the NCDs that is on the rise.^2,3^ This chronic metabolic disease is classically characterised by elevated blood glucose levels. There are numerous categories of DM, but the two main subtypes include diabetes mellitus Type 1 (DMT1) and diabetes mellitus Type 2 (DMT2).^4^

Diabetes mellitus Type 1 results from the destruction of beta cells in the pancreas, typically because of an autoimmune process and consequently patients suffer from insufficient or absent insulin levels. The onset of DMT1 increases gradually from birth and peaks between the ages of four to six years and again from 10 to 14 years, with 45% of children presenting before the age of 10 years.^4^ Diabetes mellitus Type 2 is more insidious with an imbalance of insulin levels and insulin sensitivity, resulting in a functional deficit of insulin and insulin resistance. The onset of DMT2 is typically later in life, although obesity in adolescents has led to a rise in DMT2 in younger population groups.^4^

The International Diabetes Federation (IDF) estimates that 10.5% of the global adult population (age 20–79 years) were living with DM in 2021 with almost half of them being unaware of their condition and three in four adults with DM residing in low to middle-income areas. The IDF projects that one in eight adults will be living with DM by 2045, an increase of 45%, with the most significant increase in populations from low to middle-income areas.^4,5^ The vast majority (over 90%) of people with DM have DMT2 which is driven by demographic, socio-economic, environmental and genetic factors. Contributing factors include urbanisation, decreasing levels of physical activity, increasing overweight and obesity, and an ageing population.^5^

The symptoms of DM may include polyuria, polydipsia, polyphagia, unintentional weight loss, refractive errors, paraesthesia of the hands or feet, fatigue or lethargy, poor wound healing and increased susceptibility to infections. In addition to this, DMT1 may also experience nausea and vomiting and symptoms progress rapidly in just a few weeks or months. Conversely, symptoms of DMT2 are more insipid, typically developing over several years and may go unnoticed.^6^ If left untreated, the potential complications of DM include microvascular, macrovascular and neuropathic disorders. The severity and duration of poorly controlled DM play a role in the development of microvascular and macrovascular complications, including nephropathy, retinopathy and atherosclerotic cardiovascular disease, particularly when DM is coupled with other comorbidities such as dyslipidaemia and hypertension.^7^ Roughly two-thirds of individuals with DM are at risk of succumbing to a myocardial infarction or cerebrovascular accident. In the case of DMT2, elevated fasting glucose levels exceeding 5.6 mmol/L significantly contribute to atherosclerotic cardiovascular disease risk, and cardiovascular complications can manifest even before overt hyperglycaemia sets in. Diabetes mellitus is also a leading cause of limb amputations because of vasculopathy and neuropathy.^4^

The economic strain on countries, healthcare systems, individuals with DM and their families is significant. The financial burden associated with DM has a substantial impact on global health spending, accounting for 11.5% of the total global health expenditure. The IDF estimates the cost of DM in 2021 at 966 billion USD, which represents a 316% increase over 15 years from 232 billion USD in 2007. While part of this increase can be attributed to improved data quality and collection, the IDF projects a conservative estimate of global DM-related health expenditure to reach 1.03 trillion USD by 2023 and 1.05 trillion USD by 2045.^3^ An estimated 6.7 million adults aged 20–79 years succumbed to DM or its complications in 2021. This accounts for 12.2% of global deaths in this age group from all causes. Notably, approximately one-third (32.6%) of all DM-related deaths occur in individuals of working age (under 60), constituting 11.8% of total global deaths in this demographic.^3^ It is therefore imperative to take preventative measures, identify predisposing factors and provide an early diagnosis and adequate treatment to avoid complications and the associated morbidity and mortality.

In Africa, it is estimated that more than one in two people (54%) living with DM are undiagnosed. Africa also has the second lowest DM-related expenditure (13 billion USD), accounting for 1% of global expenditure. Namibia is a developing country in sub-Saharan Africa and stands out as one of the most unequal countries globally, with a Gini coefficient of 59.1 in 2015, second only to South Africa. With an estimated population of 2.53 million (2021), the disparities in economic opportunities and access to services are significant and growing across different geographical regions.^8^ The IDF estimates the prevalence of DM for Namibia in 2021 as 5.5% (1 in 18) in the adult population (ages 20–79 years) with an age-adjusted comparative DM prevalence at 6.7% and the proportion of undiagnosed DM at 66.1%.^5^ Despite a high prevalence of undiagnosed DM in low and middle-income countries, limited studies have explored risk factors of undiagnosed DM in sub-Saharan Africa.

### Risk factors that may lead to the development of diabetes mellitus

Non-modifiable risk factors for developing DM include a family history of DM, race or ethnic background, age and a history of having gestational DM. Modifiable risk factors include excess weight, physical inactivity, raised blood pressure and cholesterol levels, smoking, unhealthy diet, heavy alcohol consumption, excessive and long-term stress and poor sleeping habits.^1,9^ In addition to this, the Centers for Disease Control and Prevention (CDC) further suggest prediabetes as a risk factor for developing DM.^6^ Prediabetes is defined as a state of hyperglycaemia with blood glucose levels that are higher than normal, but below the DM threshold.^10^

The American Diabetes Association (ADA), an International Expert Committee (IEC) and the World Health Organization (WHO) all acknowledge a fasting plasma glucose (FPG) ≥ 7.0 mmol/L (126 mg/dL) and a 2-h oral glucose tolerance test (OGTT) ≥ 11.1 mmol/L (200 mg/dL) as diagnostic indicators for DM.^11^ Additionally, the FPG and OGTT can be compared to an haemoglobin A1c (HbA1C) level in terms of sensitivity and specificity for DM detection; an HbA1C ≥ 48 mmol/mol (or 6.5%) is also diagnostic of DM.^11,12^ The Integrated African Health Observatory and the WHO African Region provide additional guidelines and suggest a random blood glucose (RBG) ≥ 11.1 mmol/L (200 mg/dL) is suggestive of DM.^13^

### Importance of screening

In the context of resource-constrained environments, the implementation of widespread screening for DM or prediabetes presents a significant challenge because of the scarcity of available resources, including financial constraints, limited medical facilities and a shortage of healthcare professionals. This necessitates a pragmatic and cost-effective approach to screening, with RBG testing emerging as a viable solution because of its simplicity and affordability.^14^ Engelgau et al. demonstrated the feasibility and effectiveness of RBG testing in identifying individuals with undiagnosed DM in resource-limited settings. The study emphasised the potential of RBG testing as a practical and cost-effective approach to DM screening in such environments.^15^ Furthermore, Bowen et al. evaluated the diagnostic accuracy of RBG for the detection of DM and found that RBG testing had reasonable sensitivity and specificity, particularly in identifying individuals with undiagnosed DM.^14^ This evidence supports the use of RBG testing as a practical means of screening in community settings with minimal resources, enabling timely interventions and optimal allocation of limited healthcare resources.

### Aim

We aimed to identify and describe the risk factors for the development of diabetes within a sample of community members in Windhoek, Namibia.

## Research methods and design

### Study design

An observational analytical cross-sectional study design was used to collect data through direct measurements of participants’ age, height, weight, abdominal girth, RBG levels, total lipid counts and blood pressures.

### Setting

Data were collected as part of a community health outreach programme in Otjomuise, an informal peri-urban settlement on the outskirts of north-western Windhoek, Namibia. Otjomuise Township constitutes one of 31 townships within the City of Windhoek. This township is home to a diverse population, reflecting various indigenous groups present across the country, including Wamboes, Damaras, Coloureds and Kavangos, among others. Otjomuise emerged in response to the influx of immigrants from rural areas and other towns into the city.^16^ Namibia contends with a variety of socioeconomic challenges, including persistent issues such as poverty, hunger, high levels of unemployment and societal inequalities, exacerbated by factors such as lagging human capital and poor access to basic services.^8,17^ Namibia has an estimated population of 2.6 million and an unemployment rate of 20%.^8,18^

### Study population and sampling strategy

Convenience sampling was adopted to enlist participants from the Otjomuise community. A mobile and temporary screening station was erected within the community for a 2-day period, and information about free screening services for community members spread rapidly through word-of-mouth. Community members over the age of 18 and who provided written consent for their demographic and health screening data to be recorded were included in the study. The final sample size was 342 (*N* = 342) participants.

### Data collection

Data were collected over a 2-day period using a Patient Profile and Health Screening Questionnaire, adapted from the WHO Standard STEPS instrument. Demographic data were collected which included age, gender, highest level of education and employment status. A focussed case history was taken, and questions regarding knowledge and attitudes of risk factors for NCDs were asked. Physical and financial access to healthcare services were assessed and physical screenings were conducted which included three separate blood pressure readings, a RBG, total cholesterol, height, weight and abdominal girth.

**FIGURE 1:**
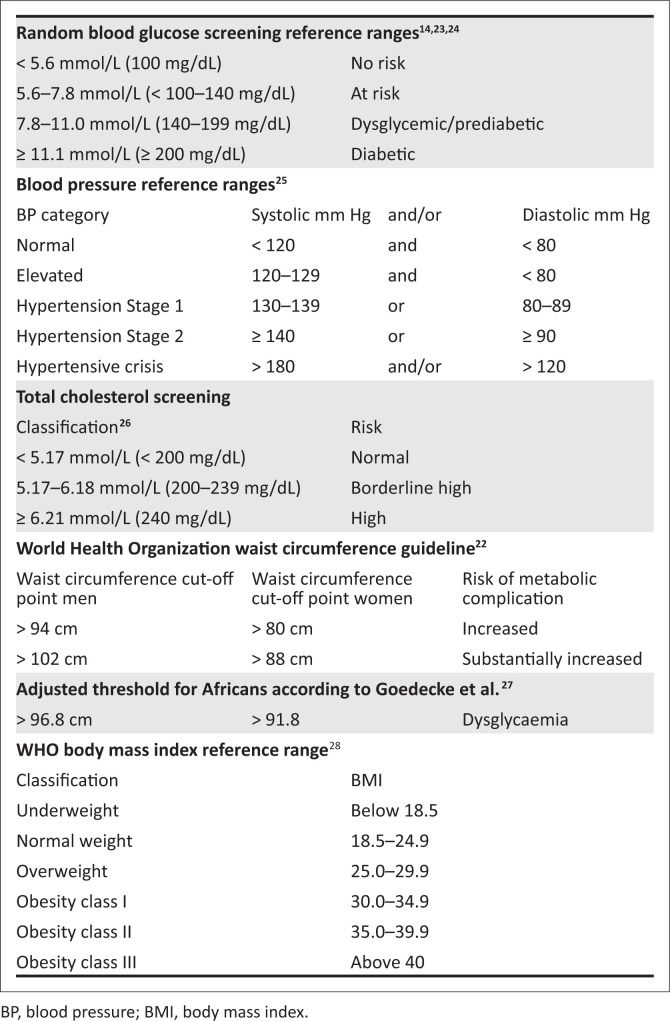
Reference ranges for random blood glucose, blood pressure, cholesterol, waist circumference and body mass index.

A microlife^®^ A150 AFIB electronic blood pressure monitor with stroke risk detection was used to assess blood pressure.^19^ To ensure accuracy, participants were seated in a chair with back support, legs uncrossed and both feet flat on the floor for a minimum of 5 min before the reading was recorded.^20^ Blood pressure measurements were taken with the arm raised to the level of the heart, and both the systolic and diastolic readings were recorded. Random blood glucose and total cholesterol were assessed using the CardioChek Plus and Unistik Touch Safety Lancets.^21^ Weight, height and abdominal girth were measured using a microlife^®^ diagnostic scale WS 80-N, stadiometer and measuring tape respectively. Abdominal girth was measured according to the WHO guidelines, at the midpoint between the lower margin of the last palpable rib and the top of the iliac crest, using a non-elastic tape.^22^ All equipment underwent calibration prior to the screening to ensure precision. Figure 1 outlines the reference ranges for RBG, blood pressure, cholesterol, waist circumference and body mass index (BMI) used in this study.

### Data analysis

The raw data were entered into an Excel® spreadsheet and subjected to descriptive analysis.

### Ethical considerations

Ethical approval to conduct this study was obtained from the University of Johannesburg Research Ethics Committee (REC-1985-2023). Written informed consent was obtained from community members to participate in the study, and all data were anonymised to ensure confidentiality of the participants.

## Results

### Demographics

Table 1 summarises the demographic profile of participants.

**TABLE 1:** Age and gender profile.

Age (years)	Sample gender and age group				
	Occurrence (*N* = 342)		Percentage		
	Male	Female	Male	Female	Total
18–29	28	54	8.2	15.8	24.0
30–39	19	50	5.6	14.6	20.2
40–49	24	62	7.0	18.1	25.1
50–59	22	48	6.4	14.0	20.5
60–69	16	13	4.7	3.8	8.5
70–79	2	1	0.6	0.3	0.9
80–89	0	3	0.0	0.9	0.9

**Total**	**111**	**231**	**32.5**	**67.5**	**100.0**

### Random blood glucose levels

Table 2 summarises the participants RBG levels.

**TABLE 2:** Random blood glucose measurements.

Random blood glucose screening	Occurrence			Percentage		
Reference ranges^14,23,24^	Men (*n* = 111)	Women (*n* = 231)	Total (*N* = 342)	Men	Women	Total
< 5.6 mmol/L (100 mg/dL)	33	76	109	29.7	32.9	31.9
5.6–7.8 mmol/L (< 100–140 mg/dL)	57	124	181	51.4	53.7	52.9
7.8–11.0 mmol/L (140–199 mg/dL)	17	22	39	15.3	9.5	11.4
≥ 11.1 mmol/L (≥ 200 mg/dL)	3	5	8	2.7	2.2	2.3
Unknown	1	4	5	0.9	1.7	1.5

*Source*: Reference ranges adapted from Bowen ME, Xuan L, Lingvay I, Halm EA. Random blood glucose: A robust risk factor for type 2 diabetes. J Clin Endocrinol Metab. 2015;100(4):1503–1510. https://doi.org/10.1210/jc.2014-4116; Bowen ME, Xuan L, Lingvay I, Halm EA. Performance of a random glucose case-finding strategy to detect undiagnosed diabetes. Am J Prev Med. 2017;52(6):710–716. https://doi.org/10.1016/j.amepre.2017.01.023; and Susairaj P, Snehalatha C, Raghavan A, et al. Cut-off value of random blood glucose among Asian Indians for preliminary screening of persons with prediabetes and undetected type 2 diabetes defined by the glycosylated haemoglobin criteria. J Diabetes Clin Res. 2019;1(2):53–58. https://doi.org/10.33696/diabetes.1.009

### Blood pressure

Each participant had three separate blood pressure measurements taken at different points during the screening process, allowing for a comprehensive assessment of the individual’s blood pressure and ensuring accuracy by minimising the influence of momentary fluctuations. Table 3 summarises the measured blood pressures.

**TABLE 3:** Blood pressure.

Blood pressure reference ranges^25^				Occurrence (average of 3 BP recordings)			
BP category	Systolic mm Hg	and/or	Diastolic mm Hg	Male (*n* = 111)	Female (*n* = 231)	Total (*N* = 342)	Total %
Normal	< 120	and	< 80	25	70	95	27.8
Elevated	120–129	and	< 80	15	35	50	14.6
Hypertension Stage 1	130–139	or	80–89	34	72	106	31.0
Hypertension Stage 2	≥ 140	or	≥ 90	32	54	86	25.1
Hypertensive crisis	> 180	and/or	> 120	5	0	5	1.5

*Source*: Reference ranges adapted from the American Heart Association. Understanding blood pressure readings [homepage on the Internet]. American Heart Association; 2024. Available from: https://www.heart.org/en/health-topics/high-blood-pressure/understanding-blood-pressure-readings

BP, blood pressure.

### Cholesterol levels

A random total cholesterol measurement was recorded for each participant. Table 4 summarises the total cholesterol measurements.

**TABLE 4:** Total cholesterol measurements.

Total cholesterol screening	Occurrence			Percentage		
Classification^26^	Men (*n* = 111)	Women (*n* = 231)	Total (*N* = 342)	Men	Women	Total
< 5.17 mmol/L (< 200 mg/dL)	85	162	247	76.6	70.1	72.2
5.17–6.18 mmol/L (200–239 mg/dL)	18	49	67	16.2	21.2	19.6
≥ 6.21 mmol/L (240 mg/dL)	7	16	23	6.3	6.9	6.7
Unknown	1	4	5	0.9	1.7	1.5

*Source*: Reference ranges adapted from Johns Hopkins Medicine. Lipid panel [homepage on the Internet]. www.hopkinsmedicine.org; 2023. Available from: https://www.hopkinsmedicine.org/health/treatment-tests-and-therapies/lipid-panel

### Waist circumferences and body mass indexes

Waist circumference was measured according to the WHO Waist Circumference and Waist-Hip Ratio guideline.^22^ Table 5 summarises the waist circumferences for participants in centimetres.

**TABLE 5:** Waist circumference measurements.

Variables			Occurrence			Percentage		
Waist circumference cut-off point men	Waist circumference cut-off point women	Risk of metabolic complication	Men (*n* = 111)	Women (*n* = 231)	Total (*N* = 342)	Men	Women	Total
**World Health Organization waist circumference guideline^22^**								
> 94 cm	> 80 cm	Increased	6	60	66	5.4	26.0	19.3
> 102 cm	> 88 cm	Substantially increased	12	81	93	10.8	35.1	27.2
**Total**			**18**	**141**	**159**	**16.2**	**61.0**	**46.5**
**Adjusted threshold for Africans according to Goedecke et al.^27^**								
> 96.8 cm	> 91.8	Dysglycaemia	15	53	68	5.4	26.0	19.9

*Source*: Waist circumference as adapted from the World Health Organisation. Waist circumference and waist-hip ratio [homepage on the Internet]. 2008. Available from: https://iris.who.int/bitstream/handle/10665/44583/9789241501491_eng.pdf?sequence=1. Adjusted threshold for Africans adapted from Goedecke JH, Nguyen KA, Kufe C, et al. Waist circumference thresholds predicting incident dysglycaemia and type 2 diabetes in Black African men and women. Diabetes Obes Metab. 2022;24(5):918–927. https://doi.org/10.1111/dom.14655

Each participant’s height and weight measurements were recorded, enabling the calculation of their BMI. Table 6 indicates the participants calculated BMIs.

**TABLE 6:** Body mass index classification.

WHO BMI reference range^28^		Occurrence			Percentage		
Classification	BMI	Men (*n* = 111)	Women (*n* = 231)	Total (*N* = 342)	Men	Women	Total
Underweight	Below 18.5	13	15	28	11.7	6.5	8.2
Normal weight	18.5–24.9	68	90	158	61.3	39.0	46.2
Overweight	25.0–29.9	23	65	88	20.7	28.1	25.7
Obesity class I	30.0–34.9	6	33	39	5.4	14.3	11.4
Obesity class II	35.0–39.9	0	21	21	0.0	9.1	6.1
Obesity class III	Above 40	1	6	7	0.9	2.6	2.0
Unknown		0	1	1	0.0	0.4	0.3

*Source*: BMI reference range adapted from the World Health Organization. A healthy lifestyle – WHO recommendations [homepage on the Internet]. World Health Organization; 2010. Available from: https://www.who.int/europe/news-room/fact-sheets/item/a-healthy-lifestyle---who-recommendations

BMI, body mass index.

A further analysis of the above data sets allowed us to identify risk factors that are commonly linked to the development of diabetes. This revealed that 55.8% of participants were aged ≥ 40 years, 66.7% had a RBG ≥ 5.6 mmol/L, 57.6% were hypertensive, 26.3% had a total cholesterol ≥ 5.17 mmol/L, 19.9% had an abnormal waist circumference and 45.3% had a BMI > 25.0 Table 7 summarises these findings.

**TABLE 7:** The risk factors identified (*N* = 342).

The risk factors identified	Men	Women	Total	%
Age ≥ 40	64	127	191	55.8
Education < less than high school	83	161	244	71.3
Unemployment	32	64	96	28.1
RBG of ≥ 5.6 mmol/L	77	151	228	66.7
Hypertension	71	126	197	57.6
Total cholesterol > 5.17 mmol/L	25	65	90	26.3
Waist circumference (men > 96.8 cm; women > 91.8 cm)	15	53	68	19.9
BMI > 25.0	30	125	155	45.3

BMI, body mass index; RBG, random blood glucose.

The study revealed 23 participants (6.7%) did not display any of the identified risk factors, while those that had one risk factor accounted for 16.7% (*n* = 57). The majority of participants (24.9%; *n* = 85) had two risk factors, followed by 21.1% (*n* = 72) having three risk factors and 17.3% (*n* = 59) having four of the six risk factors. Those with five risk factors represented 7.6% (*n* = 26) of the sample group, with 5.8% (*n* = 20) displaying all six risk factors. Table 8 represents the risk factor distribution for the sample group.

**TABLE 8:** Distribution of risk factors (*N* = 342).

Number of risk factors distribution		
Number of risk factors	Number of participants	Percentage of sample group
0	23	6.7
1	57	16.7
2	85	24.9
3	72	21.1
4	59	17.3
5	26	7.6
6	20	5.8

**Total**	**342**	**100.0**

## Discussion

Our study aimed to identify and describe risk factors for the development of DM in a sample group of community members in Windhoek, Namibia. In Namibia, 59.8% of the population are reported to be between the ages of 15–64 years with 3.97% that are 65 years and older, while the male-to-female ratio was 94.1:100 in 2022.^29,30^ The sample population in this study included those over the age of 18. The proportion of participants over the age of 60 was 10.2% and the male-to-female ratio was 45:100. The population in this study was conveniently sampled which is often criticised for its lack of representativeness. Because of resource and logistical constraints and the feasibility of accessing a truly representative sample, the focus of the study was on a small community in Windhoek, with limited data on the demographics of that community. Advancing age is a major risk factor for DM.^31,32^ According to the CDC’s National Diabetes Statistics Report, the percentage of adults with DM increased with age.^33^ Those aged 45–65 years were most likely to receive a diagnosis of DMT2. In order to identify risk factors for DM, it becomes crucial to begin screening this age group earlier. Those between the ages of 40–59 years made up the largest proportion of the sample at 45.6% with those aged between 18 and 39 years making up 44.2%.

It was observed that the majority of the sample population (52.9%) had a RBG level of between 5.6 and 7.8 mmol/L, while 11.4% recorded levels between 7.8 and 11.0 mmol/L, and 2.3% (*n* = 8) had a reading equal to or exceeding 11.1 mmol/L. While the diagnostic criteria for DM require more stringent testing, there is growing evidence to support the use of RBG testing for large-scale screening, indicating the benefit of detecting undiagnosed DM and prediabetes.^14,23^ Ziemer et al. were able to demonstrate that by using the RBG on a representative sample resulted in higher sensitivity and specificity for detecting undiagnosed DM compared to a similar study using the glucose tolerance test.^34^ Additionally, RBG testing has been suggested as a cost-effective method for identifying individuals at risk of developing DM and limits the need for fasting, indicating its convenience and practicality for large-scale screening.^23^

Susairaj et al. explored the effectiveness of RBG as an initial screening tool and identifying at-risk individuals that require further confirmatory diagnostic testing by examining the RBG cut-off values corresponding to HbA1c levels of 5.7% (39 mmol/mol) and 6.5% (48 mmol/mol). It was determined that RBG values of 6.3 mmol/L and 7.8 mmol/L were indicative of prediabetes and DM respectively, based on the aforementioned HbA1c thresholds.^23^

An Indian study compared RBG and corresponding oral glucose values in a large sample of individuals without a DM history. The findings suggested that for Asian Indians, an RBG of > 6.1 mmol/L during screening may warrant further definitive testing. The study also indicates that an RBG of 7.7 mmol/L gave the highest sensitivity and specificity and aligns with the 2-h plasma glucose ≥ 11.1 mmol/L criterion for diagnosing DM.^35^ This finding supports Susairaj et al. who determined a RBG of 7.8 mmol/L as a cut-off value for DM.^23^ Bowen et al. found that a single RBG level of ≥ 5.6 mmol/L is more strongly linked to undiagnosed DM compared to any single traditional risk factor and suggest the consideration of RBG as a DM risk factor.^24^ This evidence supports the use of RBG testing as a viable screening method in resource-constrained environments, where more elaborate tests may not be feasible because of limited resources and infrastructure. Moreover, the WHO has recognised the value of RBG testing in resource-constrained settings where FPG testing may not be feasible because of resource limitations.^13^

A RBG cut-off of 5.6 mmol/L (100 mg/dL) was used as multiple studies indicate that it achieves an optimal balance between sensitivity and specificity. While higher cut-off values might improve specificity, the 5.6 mmol/L (100 mg/dL) threshold ensures the early identification of a broader population at risk of developing diabetes or prediabetes.^14,23,24^ Bowen et al. demonstrated that using this threshold of 5.6 mmol/L (100 mg/dL) provided a sensitivity of 81.6% and a specificity of 78.0% for detecting undiagnosed diabetes, making RBG an effective tool for large-scale screening, particularly when fasting blood glucose (FBG) tests are impractical. This cut-off has proven effective in identifying individuals at risk before symptoms appear, enabling early intervention and reducing the likelihood of complications.^24^

Somannavar et al., in a study conducted in India, also found that a random capillary blood glucose (RCBG) cut-off of 5.6 mmol/L (100 mg/dL) was successful in identifying individuals requiring further diagnostic testing for prediabetes or diabetes. This study reinforces the use of the 5.6 mmol/L (100 mg/dL) threshold, particularly in resource-limited settings where fasting tests may not be feasible.^35^

Ziemer et al. further support the use of lower RBG cut-offs such as 5.6 mmol/L (100 mg/dL), emphasising its significant sensitivity for detecting undiagnosed diabetes. Their research showed that RBG screening had an area under the receiver operating characteristic (ROC) curve (AROC) of 0.80, underscoring its effectiveness in identifying abnormal glucose tolerance (AGT), especially in situations where immediate diagnostic testing may not be available. By adopting this lower threshold, more individuals in the early stages of dysglycaemia can be detected, facilitating earlier interventions that could prevent the progression to diabetes.^34^

Bowen et al. also highlighted that the 5.6 mmol/L (100 mg/dL) cut-off was more efficient in detecting undiagnosed diabetes compared to traditional screening guidelines, with a lower number needed to screen (NNTS) of 14, thus reducing unnecessary tests.^14^ Research conducted across various populations, including those in resource-limited environments such as India, supports the use of the 5.6 mmol/L (100 mg/dL) cut-off. Somannavar et al.’s study in a community-based setting effectively identified at-risk individuals needing further testing, which is crucial for early diagnosis and prevention efforts, particularly in high-risk populations.^35^

Based on these findings, 52.9% of our participants may be at risk of developing DM with an RBG level between 5.6 and 7.8 mmol/L. Participants with an RBG level between 7.8 and 11.0 mmol/L made up 11.4% of the group and may be at risk of undiagnosed DM while 2.3% of the group with an RBG level ≥ 11.1 mmol/L may be considered diabetic and certainly warrant additional testing and further monitoring. These findings are comparable to an IDF estimate of the prevalence of DM in the adult population (ages 20–79 years) for Namibia in 2021 as 6.7% and a proportion of undiagnosed DM at 51.7%.^36^

More than half of our participants (*n* = 192; 56.1%) may be regarded as hypertensive with an additional 14.6% recording elevated blood pressure and 1.5% in hypertensive crisis; a total of 72.2% of participants recording abnormal blood pressure. The relationship between hypertension and DMT2 as common comorbidities has been extensively studied and documented in the medical literature. A major contributor to morbidity and mortality in DM is cardiovascular disease, a risk heightened by the presence of hypertension. Petrie et al. found that hypertension was twice as high in individuals with DM compared to those without. Additionally, patients with hypertension often exhibit insulin resistance and are at greater risk of developing DM compared to normotensive individuals.^37^ Similar findings were observed in a 7-year follow-up study that analysed the pattern of blood pressure changes during the development of hypertension in patients with and without DM. The findings revealed that DM at baseline was a significant predictor of incident hypertension, independent of sex, age, BMI and familial DM. Conversely, hypertension at baseline was an independent predictor of incident DM.^38^ Emdin et al. observed that an increase of 20 mm Hg in systolic blood pressure (SBP) was linked to a 58% increased risk of developing new-onset DM, while a 10 mm Hg increase in diastolic blood pressure (DBP) was associated with a 52% higher risk of DM onset.^39^ These findings are supported by Nazarzadeh et al. who found consistent evidence to suggest that lowering blood pressure is likely to prevent the onset of new cases of DMT2.^40^ The associated risk of hypertension and DM together with the high occurrence of elevated blood pressure in the Otjomuise participants highlights the importance of screening and early detection to prevent future cardiometabolic complications.

Measuring total cholesterol when screening for the risk of DM holds significant value because of its association with DM prediction and cardiovascular risk assessment. Research has shown that lipid profiling, including total cholesterol, can enhance DM prediction beyond available dyslipidaemia metrics.^41^ According to the Institute for Quality and Efficiency in Health Care,^42^ a total cholesterol level below 5.2 mmol/L is considered ‘good’ in healthy individuals while the WHO indicates a desirable level below 5.0 mmol/L.^43^ For the purpose of this study, the reference ranges according to Johns Hopkins Medicine were used.^26^ In the Otjomuise sample group, 19.6% (*n* = 67) of participants had a random cholesterol reading between 5.17 and 6.18 mmol/L, which is considered borderline high, while 6.7% (*n* = 23) had recorded a reading of 6.21 mmol/L or more, considered high; a total of 26.3% (*n* = 90) of participants with an elevated cholesterol. Rhee et al. conducted a study that analysed the relationship between variations of total cholesterol and the risk of DMT2 development. It was found that participants with the highest variation of total cholesterol levels over a period of 4 years, had the highest incidence of DM, suggesting a relationship between fluctuations in cholesterol levels and the development of DMT2.^44^ Khil et al. reported that in patients with DM, a rise in total cholesterol levels from pre- to post-diagnosis was associated with an increased risk of cardiovascular disease, while a decrease in total cholesterol was associated with a reduced risk.^45^ While total cholesterol measurement provides a useful initial assessment, further evaluation is necessary where elevated levels are detected. The benefit of testing total cholesterol in a community screening project allows for early detection and timely interventions in a manner that is economical. This is especially important for quantifying the burden of cardiometabolic risk in a population group, particularly in underserved and resource-constrained areas.^46,47^

Waist circumference has been recognised as a good measure of abdominal fat and has been associated with an increased risk for DM, dyslipidaemia and hypertension.^48^ Waist circumference plays a critical role in assessing the risk of DM as research suggests it may be more strongly related to the development of DM than BMI.^49,50,51^ Furthermore, waist circumference has been shown to be genetically correlated with incident DMT2, indicating its significance in the pathophysiology of DM.^52^ In the Otjomuise sample group, 61% (*n* = 141) of women recorded a waist circumference in excess of 80 cm while 16.2% (*n* = 18) of men recorded a measurement in excess of 94 cm, for a total of 46.5% (*n* = 159) of the group at risk of metabolic complications. While glucose monitoring tools are minimally invasive, waist circumference is an efficient and cost-effective screening tool, and may complement blood glucose measurements in identifying individuals at risk of DM.

There is no consensus on the appropriate threshold for waist circumference in sub-Saharan Africa and the IDF recommends the use of Europid thresholds.^3^ Goedecke et al. proposed waist circumference as a predictor for dysglycaemia and DMT2 in African men and women; however, the optimal waist circumference threshold to predict dysglycaemia and DM in men was 96.8 cm for both, while for women, dysglycaemia was 91.8 cm and DM was 95.8 cm, which had lower sensitivity, but higher specificity than the IDF threshold of 80 cm.^27^ Previous African studies support these findings and suggest higher thresholds than the IDF guidelines for African men and women. These studies all agree that waist circumference thresholds should be region-specific and more prospective studies are needed to determine exactly what these thresholds are.^53,54,55^ When adjusting the waist circumference thresholds to those of Goedecke et al., 19.9% of the Otjomuise sample group may be at risk of dysglycaemia.

The BMI has consistently been identified as an independent risk factor for DM across various populations and age groups. Participants of the Otjomuise sample group that fell into the overweight category consisted of 25.7% (*n* = 88), with 11.4% (*n* = 39) in obesity class I, 6.1% (*n* = 21) in obesity class II, and 2% (*n* = 7) in obesity class III; a total of 45.3% (*n* = 155) with a higher-than-normal BMI. Gray et al. found that excess weight and obesity significantly contribute to the development of DMT2 and its complications in both men and women. The study revealed that individuals in the overweight category (BMI 25.0–29.9) faced an increased risk of developing DM, with a 30% greater risk for men and a 10% greater risk for women. Those in obesity class I (BMI 30.0–34.9) have a 100% higher risk of DM compared to those with a normal BMI and for those in obesity class III (BMI ≥ 40), the odds of developing DM increase by 150% for women and 180% for men.^56^ Similarly, Gupta and Bansal noted a higher probability of individuals being prediabetic or diabetic among those that are overweight or obese while Chen et al. noted these findings to be more pronounced in younger adults.^57,58^ Polemiti et al. further identified a positive association between pre-diagnosis BMI and the occurrence of vascular complications, primarily influenced by microvascular complications including kidney disease and neuropathy. Interestingly, a reduction in BMI shortly after a DM diagnosis was associated with a decreased risk of microvascular complications, kidney disease and neuropathy.^59^ In addition, Gray et al. highlight the importance of weight loss as a preventative measure for complications associated with DM and potentially slowing down progression of prediabetes to DM.^56^ There is no consensus on the waist circumference threshold for the African population, and until such time that there is, BMI may be a more reliable tool for assessing the risk of DM.

In summary, in the Otjomuise sample group, participants with an RBG ≥ 5.6 mmol/L accounted for 66.7%. While this is not diagnostic of DM or even prediabetes, this finding, coupled with the evidence of Bowen et al. who found that a single RBG level of ≥ 5.6 mmol/L was more strongly linked to undiagnosed DM compared to any single traditional risk factor, as well as the fact that estimates for Namibia’s undiagnosed DM were 51.7% as of 2021, may be supportive of initiating further testing and establishing a more diagnostic protocol for DM in these individuals.^31^ Hypertension and BMI also had a high distribution among the Otjomuise sample group with 57.6% and 45.3%, respectively. Body mass index is regarded as an independent risk factor for DM while hypertension has long been identified as a comorbid and exacerbating factor for DM. Detection of these two risk factors warrants additional screening for dysglycaemia, and even more so when coupled with an RBG ≥ 5.6 mmol/L.

In sub-Saharan Africa, DM is responsible for the highest rate of morbidity and mortality in the world, particularly among the working-age population, with 76.4% of deaths because of DM in people < 60 years in 2014. Reasons for this are attributed to late diagnosis and poor care throughout the progression of the disease.^60^ The highest proportion of participants were found to have at least two risk factors for DM which accounted for 24.9% (*n* = 85) of the Otjomuise sample group. Those with three or more risk factors accounted for 51.8% of the group. The presence of half of the identified risk factors (three out of six) in more than half of the sample group may be suggestive of the Namibian proportion of 51.7% with undiagnosed DM.

These findings have important implications for public health interventions in the management of cardiovascular and diabetes risks within the community. The identification of raised RBG, hypertension and abnormal BMI within the sample population creates a demand for the formulation of targeted strategies that address the coinciding metabolic and cardiovascular disease risks. Firstly, early detection and screening are critical components of managing these conditions. The fact that 66.7% of participants had RBG levels ≥ 5.6 mmol/L, besides the increased rates for hypertension and BMI, indicates the need for organised systematic community-based screening for such conditions. Using RBG as a primary screening tool, as demonstrated by the sensitivity and specificity of the 5.6 mmol/L cut-off, allows for early identification of at-risk individuals, particularly in resource-constrained environments where fasting tests may not be feasible. Public health interventions should focus on scaling up such screening efforts, especially in underserved areas where healthcare access may be limited. This can help identify individuals with undiagnosed diabetes or prediabetes earlier, allowing for timely interventions that can prevent disease progression.

Secondly, the integration of lifestyle modification programmes integration of lifestyle modification programmes targeting modifiable risk factors such as hypertension, physical inactivity and obesity is essential. The high occurrence of elevated BMI and waist circumference in the study population suggests that lifestyle interventions focussed on weight reduction, increased physical activity and improved dietary habits could play a critical role in reducing both diabetes and cardiovascular risks. Public health campaigns that promote physical activity, particularly in the 40–59 age group, where diabetes risk is highest, could be a key intervention. Additionally, patient education programmes that emphasise the importance of maintaining a healthy weight, controlling blood pressure and regularly monitoring glucose levels are crucial.

Hypertension management should be a priority in any public health strategy aimed at managing diabetes risk. As noted, hypertension was prevalent in 57.6% of the sample, and the relationship between hypertension and diabetes has been well-documented. Blood pressure control interventions, such as promoting medication adherence, regular monitoring and lifestyle modifications, could significantly reduce the risk of cardiovascular complications and the onset of diabetes. Additionally, public health initiatives that integrate hypertension management with diabetes screening could create more comprehensive healthcare models.

Finally, the study’s findings point to the need for tailored interventions based on regional and demographic factors. With the majority of participants falling into the 40–59 age group and nearly half of the women recording waist circumferences above 80 cm, interventions must be designed with these demographic characteristics in mind. Waist circumference has been shown to be a stronger predictor of diabetes than BMI in some populations, suggesting that waist circumference measurements should be incorporated into routine screenings. Given the unique characteristics of the Namibian population, region-specific thresholds for waist circumference and other risk factors may need to be established.

### Limitations

Possible limitations of the study include selection bias as the participants were not randomly selected, but rather conveniently sampled, which is often criticised for its lack of representativeness. The accuracy of RBG testing may influence measurement bias without a standardised protocol for testing RBG. The limited scope of screening measures is a limitation as only the RBG was used while more reliable measures such as FPG, OGTTs, and HbA1c measurement may be used to substantiate any findings. The study acknowledges the challenges of implementing large-scale community screening in resource-constrained areas and therefore the RBG was used. Additionally, the relatively small sample size limits the generalisability of the findings. Larger sample sizes in different African contexts are required to better quantify the prediabetic population.

During the community screening engagement, certain risk factors such as family history, smoking and physical inactivity were not included. This was primarily because of time constraints, language barriers and the logistical challenges of gathering comprehensive personal and lifestyle data in a large-scale community setting. Additionally, the focus of the screening was on obtaining immediate, measurable data (such as RBG, blood pressure, and cholesterol levels) to identify individuals at risk of cardiovascular and metabolic conditions. Collecting more detailed behavioural and genetic information would have required more in-depth interviews and potentially discouraged participation because of the added time and complexity. While these factors are important for a comprehensive risk assessment, they were omitted to streamline the process and ensure maximum participation and efficiency in the screening.

Addressing these limitations and considerations is essential for designing effective and sustainable screening programmes for prediabetes and DM in resource-constrained communities. Collaborative efforts involving healthcare practitioners, policymakers and community members are needed to ensure the success of such initiatives.

## Conclusion

The WHO provides clear diagnostic criteria for DM; however, emerging evidence indicates that recognised risk factors may play a crucial role in the early detection of prediabetes and DM. Given the gradual onset of symptoms of DM and the severity of its consequences, it is critical to establish effective, easily implementable and cost-effective measures for large-scale community screening. In line with the existing literature, our results support the view that there are a significant number of community members that are unaware that they have risk factors which, if left unattended, will place them at higher risk for the development of diabetes.

In conclusion, this study underscores the urgent need for early detection, lifestyle modifications and integrated care strategies to manage diabetes and cardiovascular risks in Namibia. The high occurrence of elevated RBG, hypertension and BMI highlights the necessity of comprehensive community health interventions focussed on early screening and education. While RBG testing has limitations, it remains a practical and cost-effective approach for large-scale screenings in resource-limited settings. Expanding its use, alongside public health initiatives targeting modifiable risk factors such as obesity, physical inactivity and hypertension, will be vital in reducing the future burden of diabetes and its complications. Further research with larger, more representative samples is essential to better understand the prediabetic population in different African contexts and improve public health strategies.
